# Nrf2 protects stellate cells from Smad-dependent cell activation

**DOI:** 10.1371/journal.pone.0201044

**Published:** 2018-07-20

**Authors:** Vincenzo Prestigiacomo, Laura Suter-Dick

**Affiliations:** 1 University of Applied Sciences Northwestern Switzerland, School of Life Sciences, Muttenz, Switzerland; 2 University of Basel, Department of Pharmaceutical Sciences, Basel, Switzerland; National Institutes of Health, UNITED STATES

## Abstract

Hepatic stellate cells (HSC) orchestrate the deposition of extracellular matrix (ECM) and are the primary effector of liver fibrosis. Several factors, including TGF-β1, PDGF and oxidative stress, have been shown to trigger HSC activation. However, the involvement of cellular defence mechanisms, such as the activation of antioxidant response by Nrf2/Keap1 in the modulation of HSC activation is not known. The aim of this work was to elucidate the role of Nrf2 pathway in HSC trans-differentiation involved in the development of fibrosis. To this end, we repressed Nrf2 and Keap1 expression in HSC with specific siRNAs. We then assessed activation markers, as well as proliferation and migration, in both primary and immortalised human HSCs exposed to Smad inhibitors (SB-431542 hydrate and SB-525334), TGF-β1 and/or PDGF. Our results indicate that knocking down Nrf2 induces HSC activation, as shown by an increase in αSMA-positive cells and by gene expression induction of ECM components (collagens and fibronectin). HSC with reduced Nrf2-levels also showed an increase in migration and a decrease in proliferation. We could also demonstrate that the activation of Nrf2-deficient HSC involves the TGF-β1/Smad pathway, as the activation was successfully inhibited with the two tested Smad inhibitors. Moreover, TGF-β1 elicited a stronger induction of HSC activation markers in Nrf2 deficient cells than in wild type cells. Thus, our data suggest that Nrf2 limits HSCs activation, through the inhibition of the TGF-β1/Smad pathway in HSCs.

## Introduction

Hepatic fibrosis is a scarring process in response to chronic liver injury, and it is characterized by an accumulation of fibrillar extracellular matrix (ECM) [1]. Following liver injury, hepatic stellate cells (HSCs) undergo activation, a cellular process during which HSCs trans-differentiate into myofibroblasts-like cells [1]. Activated HSC have been recognized as the responsible cells for most of the excess of ECM components in chronic liver fibrosis [1]. HSC activation is triggered by several cytokines; in particular platelet-derived growth factor (PDGF) and transforming growth factor-β1 (TGF-β1), released from platelet and Kupffer cells respectively, have been identified as the main mitogenic and pro-fibrotic mediators for HSCs [1].

Increasing evidence has shown that oxidative stress may promote fibrosis and HSC activation in the human liver and rodents [2,3]. In many cell types the transcriptional response to oxidative stress is mediated by a cis-acting element termed antioxidant response element (ARE); the nuclear factor E2-related factor 2 (Nrf2) has been identified as the most important transcription factor acting on the ARE for many genes [4–6]. In the human genome, Nrf2 regulates the transcription of more than 500 genes, most of which have a cytoprotective role [6].

A key element for the regulation of the activity of Nrf2 is the Kelch-like ECH-associated protein 1 (Keap1), which acts as a constitutive repressor of Nrf2 [4]. Under normal conditions, Nrf2 is bound to Keap1, which is an adaptor molecule for the Cullin3-based E3 ubiquitin ligase complex, leading to the degradation of Nrf2 via the by ubiquitin-proteasome pathway [5]. In this condition, Nrf2 appears as a highly unstable protein with a half-life of around 15 minutes [4]. Oxidative or electrophilic stress causes the inactivation of Keap1, resulting in Nrf2 stabilization, nuclear translocation and subsequent gene induction [5].

Among other organs, Nrf2 plays a predominant role in the liver, since it is a key regulator of the constitutive and inducible expression of some phase II and III detoxification enzymes and antioxidant proteins, such as those involved in glutathione synthesis, in primary hepatocytes and hepatocyte-like cells [7]. Several studies reported that Nrf2-knockout mice showed an exacerbated cytotoxicity to acetaminophen (APAP) as well as a strongly aggravated liver damage after treatment with CCl4 or ethanol [8–11]. In a similar study, Okawa et al. showed that Keap1-knockout mice were significantly more resistant to APAP than control animals [12]. Concordantly, experiments in mice showed an increase in nuclear translocation of Nrf2 after APAP administration [13]. In addition to its protective role, it has been demonstrated that Nrf2 regulates hepatocyte proliferation by ensuring normal insulin/IGF-1 and Notch1 signalling during liver regeneration [14,15]. Recently, we showed upregulation of both Nrf2 and Keap1 after Methotrexate- and Thioacetamide- induced fibrosis in a 3D human cell culture model, indicating a role of Nrf2 in hepatic fibrosis [16]. Nrf2 has been shown to have antifibrotic effect in liver, lung and kidney, by promoting the dedifferentiation of fibroblasts [17–19].

Many studies have been conducted on Nrf2-related effects on liver but little is known about its role on HSCs. It has been reported that Nrf2 inhibits the TGF-β1-dependent expression of fibrosis markers in a human stellate cell line [17]. Yang et al. have shown that TGF-β1 reduced the presence of Nrf2 in a rat hepatic stellate cell line [3]. This effect was dependent on the epigenetic regulation operated by the microRNA-200a. However, although these studies suggest an involvement of Nrf2 in HSC activation, studies conducted on human primary HSCs are still lacking. Here, we report the effects of Nrf2-knockdown on proliferation, migration and activation of human HSCs. Our studies demonstrate that Nrf2-knockdown induces HSC activation in both human primary and immortalised (hTERT) HSCs. Moreover, this activation can be modulated by TGF-β1, PDGF-AB and/or Smad inhibitors. These results may contribute towards studying and developing new successful and advantageous therapies for liver fibrosis.

## Material and methods

### Reagent, chemicals and antibodies

DMEM High Glucose (41965; Invitrogen), Fetal Bovine Serum (FBS) (10270; Invitrogen), Penicillin-Streptomycin (A8943; Applichem), TGF-β1 (T5050; Sigma), PDGF-AB (P8147; Sigma), SB-431542 hydrate (SB43) (S4317; Sigma), SB-525334 (SB52) (S8822; Sigma), Triton X-100 (T8787; Sigma), Bovine serum albumin (BSA) (05473; Fluka), Lipofectamine® RNAiMAX Transfection Reagent (13778030; Thermo Fisher), OptiMEM (51985034; Thermo Fisher), αSMA antibody (A5228; Sigma), secondary antibodies Alexa Fluor® 488 (A11017 and A11078; Invitrogen), DAPI (D9542; Sigma), Propidium Iodide (7109; Sigma), anti-β-Actin antibody (sc-47778; Santa Cruz Biotechnology), anti-Nqo1 antibody (Ab2346; Abcam), anti-Nrf2 antibody (16396-1-AP; Proteintech), Anti-α-Tubulin antibody (T8203; Sigma), anti-Keap1 antibody (10503-2-AP; Proteintech), Anti Mouse-AP (4760.1; Roth), Anti-Rabbit Peroxidase antibody (A6154; Sigma), Anti-Goat Peroxidase antibody (A16008; Invitrogen).

### Cell culture and treatments

Human primary HSCs were purchased from iXCells Biotechnologies, USA, (Cat. 10HU-210) or Innoprot, Spain (Cat. P10653). They were cultured in DMEM High Glucose supplemented with 10% FBS and 1% Penicillin-Streptomycin (complete DMEM) and used up to 5 passages. hTERT-HSC were kindly provided by Dr. Bernd Schnabl (UC San Diego, USA)[20] and were cultured in complete DMEM up to 12 passages. The cells were kept in the humidified incubator at 37°C with 5% CO_2_. Cell treatments were performed in serum-free medium for a maximum of 48 hours without changing the medium. Following concentration were used as indicated in the legend: 0.5–10 ng/mL TGF-β1, 1–5 ng/mL PDGF-AB, 10 μM SB43, 1 μM SB52.

### Nrf2 and Keap1 knockdown

A number of 1.9 x 10^5^ HSCs per well were seeded in 12-well plates. The cells were transfected at day zero with siPOOL5 targeting human NFE2L2 (Nrf2) (NM_001145412; siTOOLs Biotech) and human Keap1 (NM_012289; siTOOLs Biotech). Briefly, a mixture containing siRNAs and Lipofectamine was prepared in OptiMEM and added into the well at 1:10 dilution in complete DMEM, in order to achieve a final concentration of 5 nM siRNA and 2 μL Lipofectamine per well. The cells were transfected for 72 hours and then detached and used for the next experiments. Scrambled siRNAs were purchased by siTOOLs Biotech and used as negative control (indicated as siCON from now).

### Immunocytochemistry analysis

After different treatments, HSCs were fixed in 4% formaldehyde for 15 minutes, followed by permeabilization with 0.1% Triton-X-100 for 20 minutes. Blocking was performed with 1% BSA in PBS for 60 minutes, followed by incubation with primary antibody against αSMA (dilution 1:200 in blocking solution) or Nqo1 (1:500) for 90 minutes. Incubation with secondary antibody was performed for 60 minutes in blocking solution at a 1:400 dilution. All steps were conducted at room temperature. DAPI and Propidium Iodide were used to stain the nuclei.

### Click-iT EdU proliferation assay

The Click-iT® EdU Alexa Fluor® 488 Imaging Kit (C10637; Invitrogen) was used to measure the proliferation rate of the cells after treatments. After cell transfection, the cells were detached and 3 x 10^4^ cells/cm^2^ were seeded in 96-well plate format. After overnight attachment, the cells were treated with EdU for 30 hours. In a separate experiment, detached cells were seeded and immediately treated with 0.5–1 ng/mL TGF-β1, 1–5 ng/mL PDGF-AB and a mixture with 1 ng/mL TGF-β1 and 5 ng/mL PDGF-AB for 48 hours. EdU dye was added only during the last 30 hours of each experiment and detected following manufacturer’s instructions. The pictures were acquired with the Olympus Laser Confocal Scanning Microscope FV1000D spectral type (inverted microscope IX81) and analysed with ImageJ to determinate the proliferation rate.

### Gene expression analysis

To determinate the efficiency of transfection, as well as the activation profile of the HSCs, RNA from treated and untreated cells was isolated following TRIzol extraction procedure. RNA was reverse transcribed using a reverse transcriptase (Promega) and oligo dT (Qiagen) and real time PCR was performed using FastStart TaqMan Mix (Roche) and TaqMan probes from Invitrogen. Real time, Taqman qPCR was performed on selected genes (see Table 1). The following qRT-PCR Program was used: 10 minutes denaturation at 95°C, followed by 40 cycles of 15 seconds at 95°C and 1 minute at 60°C. The Ct values were assessed using the Corbett Rotorgene Analysis Software 6000, and B2M was used as an internal standard for the normalization of the fold changes of each gene of interest (GOI). Heat map for each gene and condition was obtaining by using the Heatmapper online software (http://www2.heatmapper.ca/expression/).

**Table 1:** TaqMan probes used for the research.

Gene of interest	Abbreviation	Invitrogen Ref.nr.
Beta-2-microglobulin (Housekeeping gene)	B2M	Hs00187842_m1
Actin, alpha 2, smooth muscle	ACTA2 (αSMA)	Hs00426835_g1
Activating transcription factor 3	ATF3	Hs00231069_m1
Catenin beta 1	CTNNB1	Hs00355049_m1
Collagen 1 alpha 1	COL1α1	Hs00164004_m1
Collagen 4 alpha 1	COL4α1	Hs00266237_m1
Fibronectin 1	FN1	Hs00415006_m1
Hyaluronic acid receptor	CD44	Hs01075861_m1
Kelch-like ECH-associated protein	Keap1	Hs00202227_m1
Lysyl oxidase	Lox	Hs00942480_m1
Lysyl oxidase like 2	Loxl2	Hs00158757_m1
Metalloproteinase 2	MMP2	Hs01548727_m1
Mitogen-activated protein kinase 8	MAPK8	Hs01548508_m1
Mitogen-activated protein kinase 14	MAPK14	Hs01051152_m1
NAD(P)H quinone dehydrogenase 1	Nqo1	Hs02512143_s1
Nuclear factor (erythroid-derived 2)-like 2	NFE2L2 (Nrf2)	Hs00975961_g1
PDGF receptor alpha	PDGFRA	Hs00998018_m1
PDGF receptor beta	PDGFRB	Hs01019589_m1
Snail family transcriptional repressor 1	Snail1	Hs00195591_m1

### Migration assay

The migratory capacity of HSCs was investigated using the Culture-Insert 2 Well (80209; Ibidi) according to the manufacturer's instructions. Briefly, 70 μL of 3 x 10^5^ cells/mL suspension were incubated in each chamber in serum-free medium overnight. After cell attachment, the culture insert was gently removed by using sterile tweezers, leaving a cell-free gap of approximatively 500 μm. Medium was slowly aspired and 1 mL/well of serum free DMEM medium was added. Migration of transfected HSCs was evaluated in presence of TGF-β1, PDGF-AB and SB52 inhibitor. The wound healing process was followed during 48 hours by time laps microscopy, using Olympus cellVivo incubation system with 4X magnification. Pictures were acquired every hour for a period of 48 hours. Pictures were analysed with ImageJ and migration area was calculated with MRI Wound Healing Tool (http://dev.mri.cnrs.fr/projects/imagej-macros/wiki/Wound_Healing_Tool) as previously published [21].

### ELISA

The presence of TGF-β1 in the supernatants of transfected HSCs was determined using commercial human ELISA kit (ABIN1979586; Antibodies-online GmbH), strictly following manufacturer’s instructions.

### Western blot analysis

Cells were lysed in RIPA lysis buffer (89900; Thermo Fisher) on ice. Whole extracts were prepared and proteins were quantified by using a standard Bradford assay. 30 μg of proteins were separated by SDS-PAGE on Biorad precast anykD gel (456–9033; Biorad) and then blotted onto a nitrocellulose membrane (GE10600004; Sigma). After 60 minutes blocking with Odyssey® Blocking Buffer (PBS) (927–40000, LI-COR), the membranes were incubated overnight with primary antibodies diluted in blocking buffer as following: anti-β-Actin antibody (1:500), anti-Nqo1 antibody 1:5000), Anti-α-Tubulin antibody (1:2000), anti-Keap1 antibody (1:2000). For Nrf2 detection blocking overnight was performed, followed by overnight incubation with anti-Nrf2 antibody (1:750). Horseradish peroxidase conjugated anti-mouse, anti-rabbit and anti-goat antibodies were used as secondary antibodies for 60 minutes in blocking buffer at the dilution of 1:5000. After extensive washing in PBS-T, the membranes were developed by incubating for 10 minutes in presence of BCIP and NBT in AP-buffer, and intensities quantified using Image J analysis software as previously published [22].

### Statistical analysis

Experiments with primary HSC were conducted with at least 3 batches of cells as indicated in the legend. Data were analysed using GraphPad Prism 7 (GraphPad Software, inc.) and expressed as mean values ± SD as indicating in the legend. The Student’s t-test was used for comparison between two groups. Data from three or more groups were analysed by one-way analysis of variance with Tukey's multiple comparisons test. P ≤ 0.05 was considered significant.

## Results

### Exposure to TGF-β1 suppresses gene expression of Nrf2 and related genes

**Fig 1:**
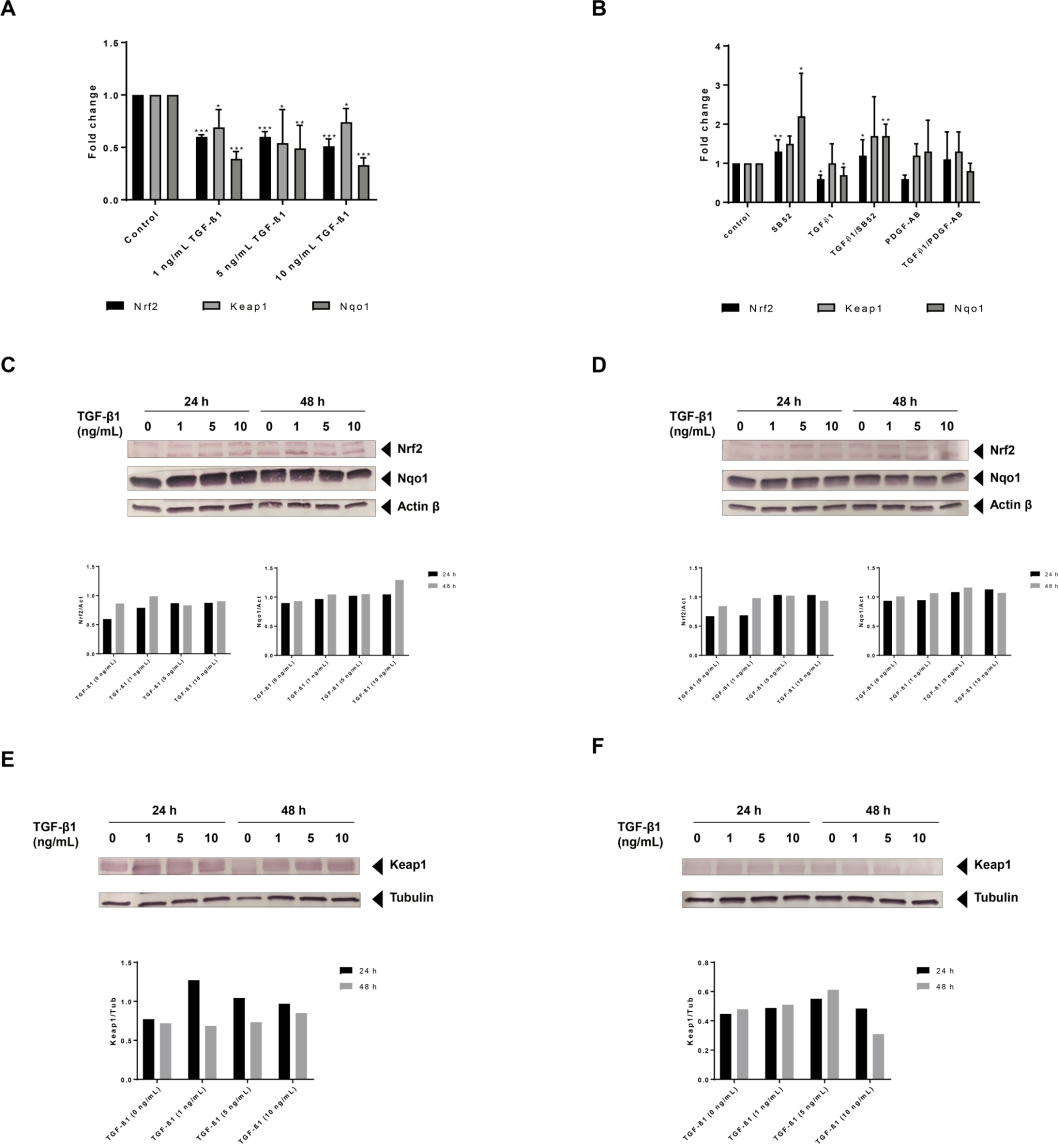
TGF-β1 suppresses mRNA expression of Nrf2 in HSCs. (A) hTERT-HSCs were exposed to 1–5 ng/mL TGF-β1 for 48 hours. mRNA was extracted using TRIzol conventional procedure and fold changes were calculated as 2^(-ΔΔCT) for each sample and control and expressed as mean fold change ± SD (N = 3). Beta-2-microglobulin (B2M) was used as reference gene for each sample. The results show a significant downregulation of Nrf2, Keap1 and Nqo1 after exposure to TGF-β1. (B) Primary HSCs were exposed to 1 ng/mL TGF-β1, 1 μM SB-525334 (SB52) and/or 5 ng/mL PDGF-AB for 48 hours. mRNA was extracted using TRIzol conventional procedure and fold changes were calculated as 2^(-ΔΔCT) for each sample and control and expressed as mean fold change ± SD (N = 3 different batches). Beta-2-microglobulin (B2M) was used as reference gene for each sample. The results show a significant downregulation of Nrf2, and Nqo1 following exposure to TGF-β1. SB52 induces upregulation of Nrf2 and Nqo1, efficiently inhibiting the TGF-β1 effect. (C-D) The protein levels of Nrf2, Nqo1 and actin beta were analysed by Western blot analysis after exposure to 0–10 ng/mL TGF-β1 in hTERT-HSC (C) and primary HSCs (D) for 24 and 48 hours. (E-F) The protein levels of Keap1 and Tubulin were analysed by Western blot analysis after exposure to 0–10 ng/mL TGF-β1 in hTERT-HSC (E) and primary HSCs (F) for 24 and 48 hours.

Based on our own and previously published data [1,16,23], we exposed the HSCs to TGF-β1 in order to induce HSC activation. At all tested TGF-β1 concentrations, gene expression levels of Nrf2, Keap1 and Nqo1 were significantly downregulated in immortalized HSC (hTERT-HSC) after 48 hours of treatment (Fig 1A). Nrf2 mRNA levels were downregulated in a concentration-dependent manner already after 24 hours (S1A Fig), while Nqo1 showed a significantly downregulation only after 48 hours of exposure (Fig 1A and S1C Fig). Downregulation of Keap1 was observed at all three concentrations of TGF-β1 and at all time points (except 1 ng/mL at 24 hours) (Fig 1A and S1B Fig). In primary HSC, TGF-β1 significantly decreased the mRNA levels of both Nrf2 and Nqo1after 48 hours of exposure, but no change in expression was observed for Keap1 (Fig 1B). The downregulation of Nrf2 and Nqo1 by TGF-β1 could be prevented by the Smad inhibitor SB52. This inhibitor led to the upregulation of the mRNA levels of both Nrf2 and Nqo1, even when applied in combination with TGF-β1, supporting a direct link between TGF-β1 stimulation and Nrf2-repression (Fig 1B).

In addition to the analysis of the mRNA levels of selected markers, we determined the protein levels of Nrf2, Keap1 and Nqo1 after exposure of HSCs to TGF-β1 (Fig 1C–1F). Both human immortalised and primary HSCs (Fig 1C and 1D), showed high basal levels of Nqo1. The down-regulation of Nrf2 mRNA caused by exposure to TGF-β1 did not lead to any significant changes in its protein levels at 24h. On the contrary, Nrf2 levels were slightly increased by 1 ng/mL TGF-β1 at 48 hours in both HSCs. An increase in the protein levels of Keap1 was observed after treatments with TGF-β1 in hTERT-HSC in a concentration dependent manner at both 24 and 48 hours exposure (Fig 1E). Lower levels of Keap1 were observed in primary HSC compared to hTERT-HSC, and a slight increase after TGF-β1 exposure was observed at 24 hours only (Fig 1F).

### Nrf2 knockdown induces stellate cell activation

**Fig 2:**
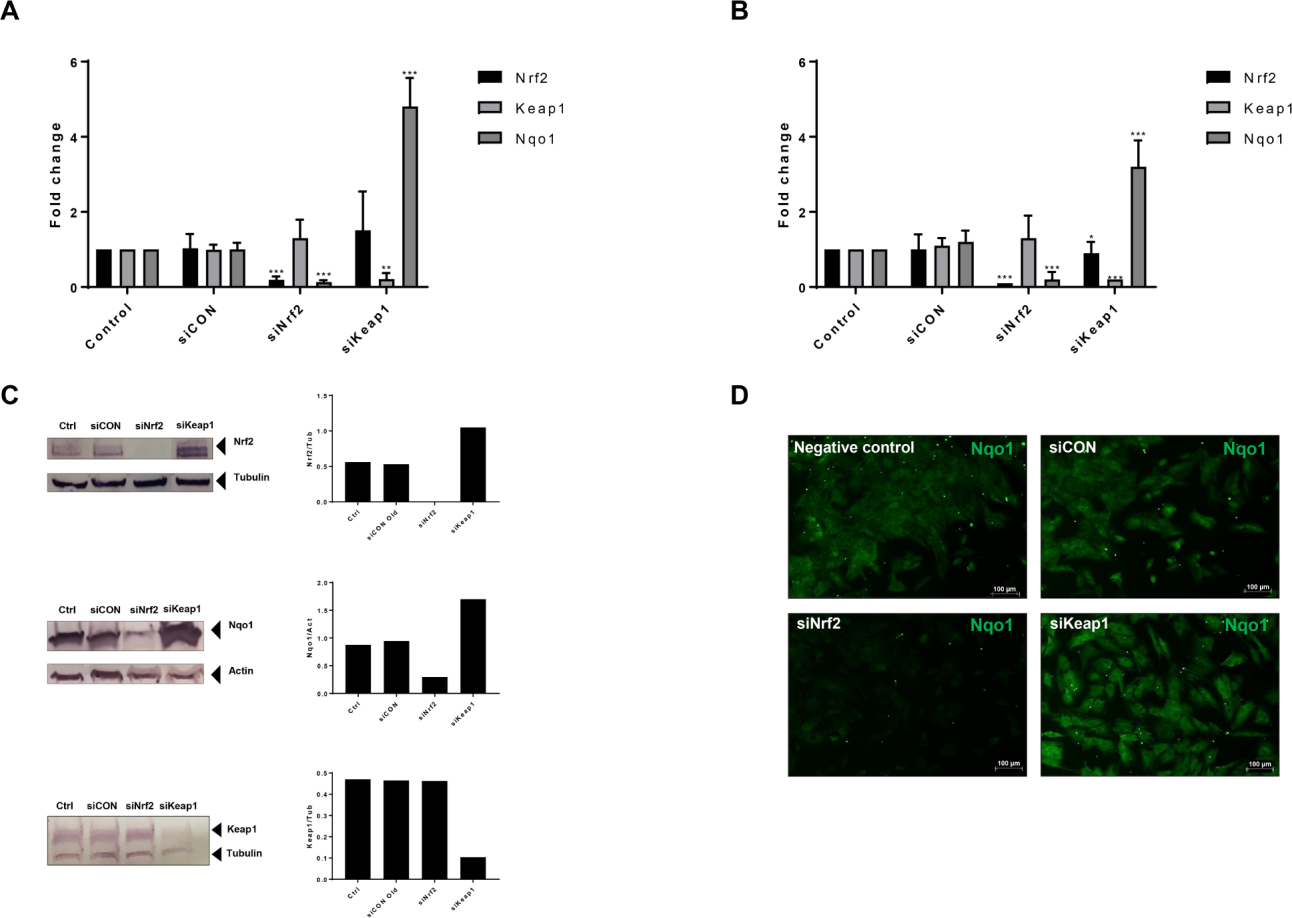
siRNA knockdown of Nrf2 pathway-related genes. (A-B) siRNAs for Nrf2 (siNrf2), Keap1 (siKeap1) and scrambled (siCON) were used to knockdown Nrf2 and Keap1 in hTERT-HSC (A) and primary HSC (B). mRNA level of Nrf2, Keap1 and Nqo1 were then analysed, showing an efficient knockdown of both Nrf2 and Keap1. Fold changes were calculated as 2^(-ΔΔCT) for each sample and control and expressed as mean fold change ± SD. *, P ≤ 0.05; **, P ≤ 0.01; ***, P ≤ 0.001 vs siCON (mean ± SD, N = 4 from independent experiment for hTERT-HSC and N = 6 different batches in primary HSC). (C) Protein levels of Nrf2, Keap1 and Nqo1 were analysed by Western blot in knockdown hTERT-HSC. Ctrl: control samples; siCON: scrambled siRNA; siNrf2: Nrf2 knockdown; siKeap1: Keap1 knockdown. Protein were extracted 72 hours after the transfection. (D) Immunocytochemistry analysis of Nqo1 in hTERT-HSC. Green: Nqo1. siCON: scrambled siRNA; siNrf2: Nrf2 knockdown; siKeap1: Keap1 knockdown.

To further investigate the role of Nrf2 in HSC activation, we performed the knockdown of Nrf2 or Keap1 on HSCs using specific siRNAs (siNrf2 and siKeap1). After 72 hours of incubation with siRNA, we could detect an efficient knockdown in both primary and immortalised HSCs: mRNA of Nrf2 and Nqo1 were significantly downregulated by 80–90% in both primary and hTERT-HSCs after siNrf2 (Fig 2A and 2B). Protein levels were visualised by western blot in hTERT-HSC, showing a successful knockdown also at the protein level (Fig 2C). In addition, immunostaining of Nqo1 in hTERT-HSC confirmed the downregulation after exposure to siNrf2 (Fig 2D). The knockdown was maintained for up to 7 days after transfection (data not shown). As expected, siKeap1 induced downregulation of Keap1 mRNA, and a concomitant upregulation of Nqo1 mRNA and protein (Fig 2A–2C). In particular, mRNA levels of Nqo1 were upregulated by 4.8- and 3.2-fold changes after siKeap1 in hTERT-HSC and primary HSC, respectively.

**Fig 3:**
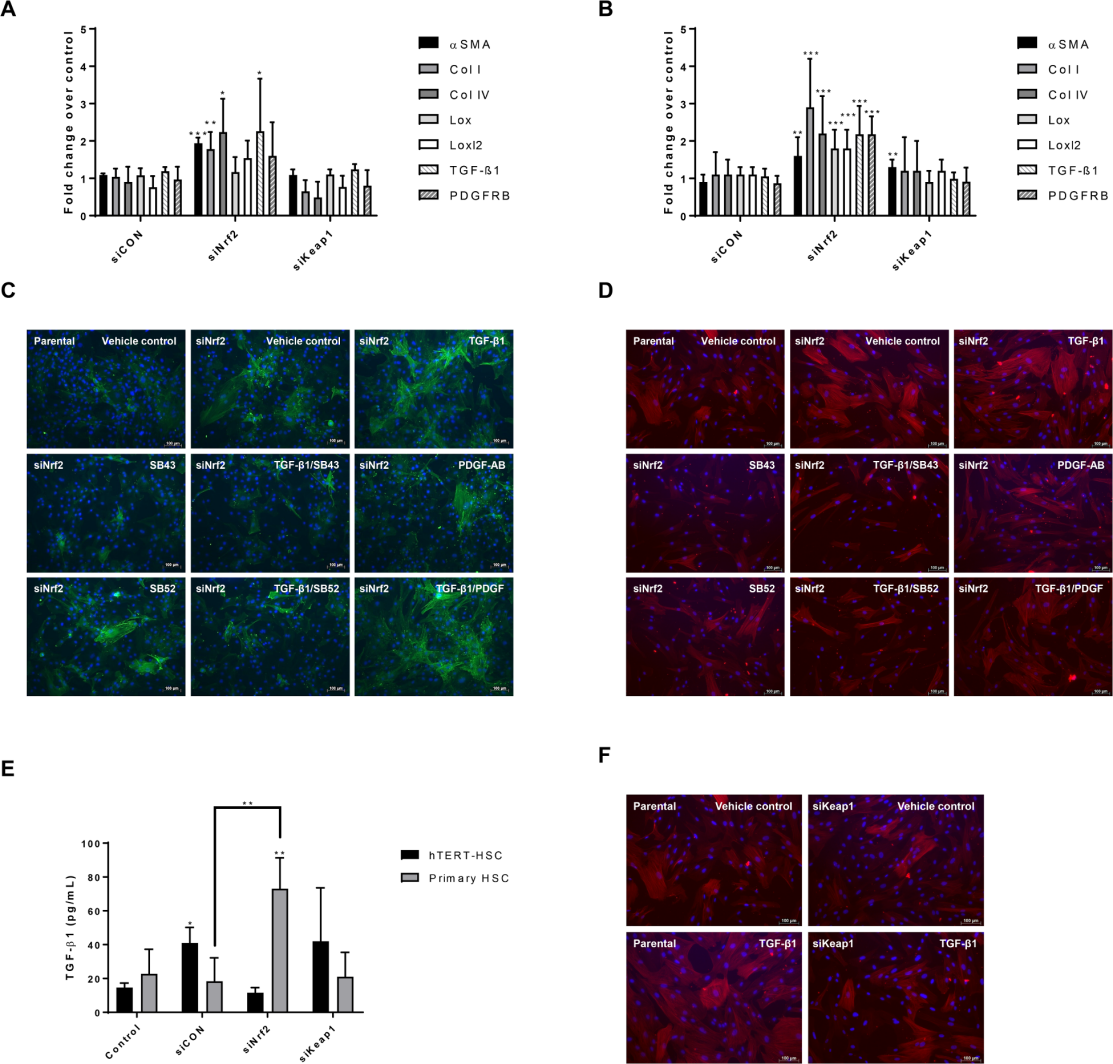
Effect of Nrf2 knockdown on HSC activation and response to stimuli. (A-B) After 72 hours knockdown with siRNAs, mRNA was extracted from hTERT-HSC cells (A) and human primary HSCs (B). Fold change for each gene of interest was calculated as 2^(-ΔΔCT) for each sample and control and expressed as mean fold change ± SD. Beta-2-microglobulin (B2M) was used as reference gene for each sample. *, P ≤ 0.05; **, P ≤ 0.01; ***, P ≤ 0.001 vs Control (mean ± SD, N = 4 from independent experiment for hTERT-HSC and N = 6 different batches for primary HSC). (C-D) hTERT-HSCs (C) and primary HSCs (D) were treated for 48 hours with TGF-β1 (1 ng/mL), SB431542 hydrate (SB43) (10 μM), SB-525334 (SB52) (1 μM) and/or PDGF-AB (5 ng/mL). After treatment, the cells were fixed and stained against αSMA (green in C and red in D) and nuclei (DAPI, blue). The results show an increase in αSMA production in Nrf2 knockdown cells and a further increase after TGF-β1 exposure. SB43 and SB52 significantly inhibited the TGF-β1-induced αSMA. Pictures taken using fluorescence microscopy. siNrf2: Nrf2 knockdown. Scale bar: 100 μm. (E) TGF-β1 release was measured in supernatant medium in both transfected hTERT and primary HSCs by ELISA. siCON: scrambled siRNA; siNrf2: Nrf2 knockdown; siKeap1: Keap1 knockdown. *, P ≤ 0.05; **, P ≤ 0.01 vs Control or siCON (mean ± SD, N = 4 from independent experiments for hTERT-HSC and N = 5 different batches for primary HSC). (F) Immunostaining of αSMA (red) and nuclei (DAPI, blue) in Keap1 knockdown primary HSCs. Knockdown cells show more resistance again TGF-β1 (1 ng/mL) induced activation compared to control cells. siKeap1: Keap1 knockdown.

Transfection of both immortalised and primary HSCs with siNrf2 caused HSC-activation determined by transcriptional induction of αSMA, Collagens I and IV, and TGF-β1 (Fig 3A and 3B, respectively). Lox, loxl2 and PDGFRB were also upregulated in human primary HSCs (Fig 3B). No significant changes were seen in both scrambled control (siCON) and siKeap1 HSCs. Secreted TGF-β1 was also significantly increased in primary HSC from 20 to 70 pg/mL after siNrf2 transfection (Fig 3E). Additional evidence for the activation of HSCs by siNrf2 is provided by the strong upregulation of αSMA and its organization into stress fibres (Fig 3C and 3D). This effect was inhibited by the Smad inhibitors SB43 and SB52, indicating an involvement of the TGF/Smad pathway. In this experimental set up, stronger inhibition was observed with SB43 rather than SB52 in both primary and immortalised HSCs. As expected, PDGF-AB did not affect αSMA production, while TGF-β1 further induced αSMA and stress fibres formation in a Smad-dependent manner, as indicated by the immunostaining in presence of TGF-β1 and/or SB inhibitors (Fig 3C and 3D). Interestingly, siKeap1 showed a protective effect against TGF-β1-induced αSMA production compared to negative control in primary HSCs, suggesting a protective role of Nrf2 against TGF-β1-induced HSC activation (Fig 3F).

### Nrf2 knockdown reduces cell proliferation and increases PDGF-induced proliferation rate in HSCs

**Fig 4:**
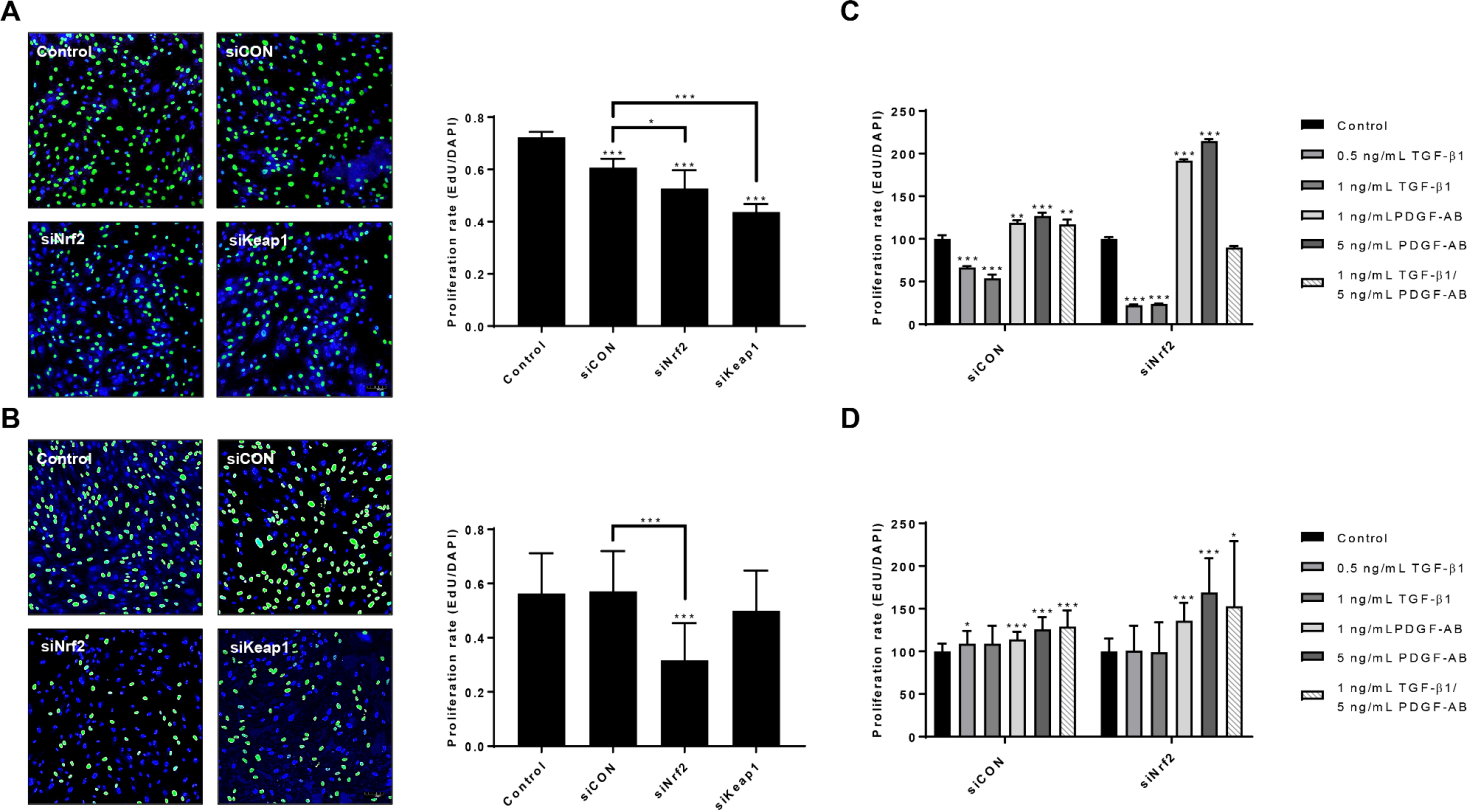
Effect of siRNAs, TGF-β1 and/or PDGF-AB on HSC proliferation. (A-B) hTERT-HSCs (A) and primary HSCs (B) were transfected for 72 hours with siNrf2, siKeap1 or siCON. Cells were then detached and plated for EdU staining as described in Materials and methods section. The pictures were taken with 10X magnification by using confocal microscopy and nuclei were counted with Image J software. siCON: scrambled siRNA; siNrf2: Nrf2 knockdown; siKeap1: Keap1 knockdown. *, P ≤ 0.05; **, P ≤ 0.01; *** P ≤ 0.001 vs control or siCON. Values are expressed as rate of proliferation (mean ± SD); N = 4 independent experiment with 5 replicates each for hTERT-HSC, and N = 6 different batches with 5 replicates each. (C-D) Proliferation rate of transfected hTERT-HSC (C) and primary HSC (D) after exposure to 0.5–1 ng/mL TGF-β1 and/or 1–5 ng/mL PDGF-AB for 48 hours. EdU was added during the last 30 hours of the experiment. Values are expressed as percentage of proliferation over each control sample. *, P ≤ 0.05; **, P ≤ 0.01; *** P ≤ 0.001 vs Control (mean ± SD, N = 4 replicates for hTERT-HSC and N = 3 from different batches with 4 replicates each for primary HSC).

To explore the proliferative capacities of the transfected HSCs, we performed an EdU proliferation assay (Fig 4). The proliferation rate was around 0.7 for parental hTERT-HSC, and around 0.6 for parental primary HSC. In both HSCs, siNrf2 significantly reduced the proliferation rate by around 30% (Fig 4A and 4B). siCON and siKeap1 did not affect the proliferation rate in the primary HSCs, while they significantly reduced the proliferation in the hTERT-HSC. Nrf2 knockdown cells showed more PDGF-AB-induced cell proliferation than siCON cells (Fig 4C and 4D). Proliferation was in fact increased by 50–100% in both siNrf2 HSCs after exposure to PDGF-AB compared to siCON HSCs where only an increase of 25% was detected. These results suggest that Nrf2 knockdown might drive the cells towards activation, making them more sensitive to stimuli. TGF-β1 did not affect the proliferation rate in primary HSCs, neither with siCON nor with siNrf2 (Fig 4D), while downregulation was measured in hTERT-HSC, with a more pronounced effect after Nrf2 knockdown (Fig 4C).

### Knockdown of Nrf2 induces cell migration in HSCs

**Fig 5:**
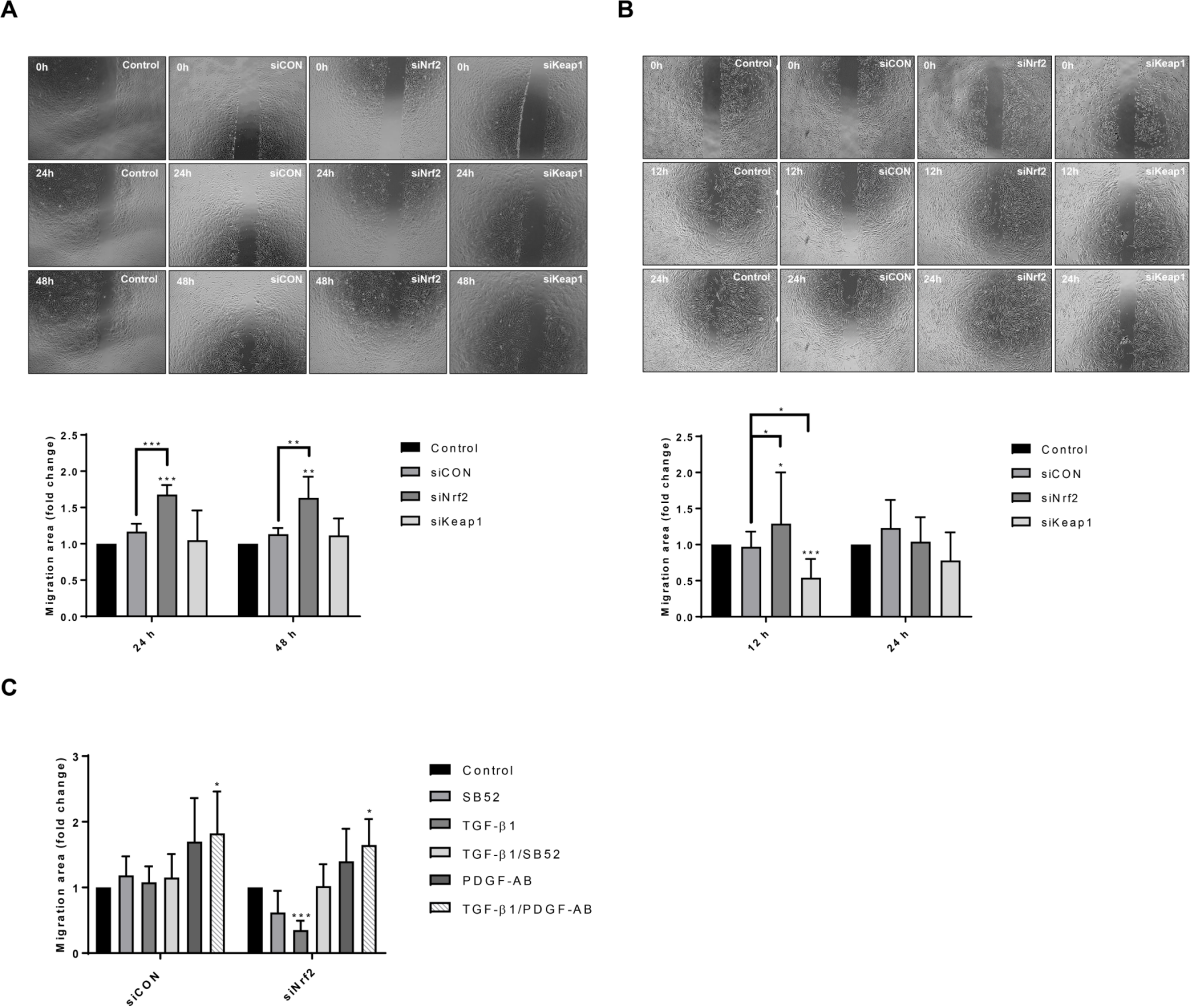
Nrf2 knockdown-dependent migration of HSCs. After 72 hours of knockdown, hTERT-HSCs (A) and primary HSCs (B) were seeded into the Culture Insert 2-Well (Ibidi). Pictures were acquired with 4X magnification every hour for a period of 48 hours using Olympus cellVivo incubation system. Pictures of the samples at 0, 24 and 48 hours are showed for hTERT-HSC (A), while 0,12 and 24 hours are showed for primary HSCs (B). Migration area was calculated with MRI wound healing tool of Image J software and expressed as fold change of the treated vs control (mean ± SD). *, P ≤ 0.05; **, P ≤ 0.01 vs Control. N = 4 independent experiments for hTERT-HSC (A) and N = 6 different batches for primary HSC (B). (C) After transfection, primry HSCs were seeded into the Culture Insert 2-Well and exposed to TGF-β1 (1 ng/mL), SB-525334 (SB52) (1 μM) and/or PDGF-AB (5 ng/mL) for 48 hours. Pictures were acquired with 4X magnification every hour for a period of 48 hours using Olympus cellVivo incubation system. Migration area at 12 hours was calculated with MRI wound healing tool of Image J software and expressed as fold change of the treated vs control (mean ± SD). *, P ≤ 0.05; ***, P ≤ 0.001 vs Control (N = 3 different batches).

Nrf2 knockdown induced a significant increase in cell motility in both hTERT-HSC and primary HSCs while Keap1 knockdown induced a decrease in the migration rate only in primary HSCs (Fig 5). In particular, siNrf2 hTERT-HSC displayed 1.5-fold higher migration than control cells at both 24 and 48 hours (Fig 5A); no changes were measured after siKeap1 and siCON. Primary HSCs showed higher migration than hTERT-HSC, with a complete repair of the wound after 24 hours (data not shown). Thus, the migration rate was calculated at 12 and 24 hours for primary HSCs (Fig 5B). Similarly, to hTERT-HSC, siNrf2-treated primary HSCs displayed higher migration than control cells at 12 hours; no significant changes were measured at 24 hours. The motility of knockdown cells was further affected by TGF-β1 and PDGF-AB (Fig 5C). In fact, transfected primary HSCs showed a PDGF-AB-induced cell migration in both siCON and siNrf2 cells with a synergistic effect of TGF-β1 and PDGF-AB (Fig 5C). TGF-β1 did not affect the migration of control cells (siCON), while a decrease in migration was displayed in the Nrf2 knockdown cells, indicating a stronger response of the cells to the cytokine. No significant effect of SB52 was observed. Similar data were obtained with hTERT-HSC (data not shown).

### Nrf2 knockdown induces stellate cells activation in a Smad-dependent manner

**Fig 6:**
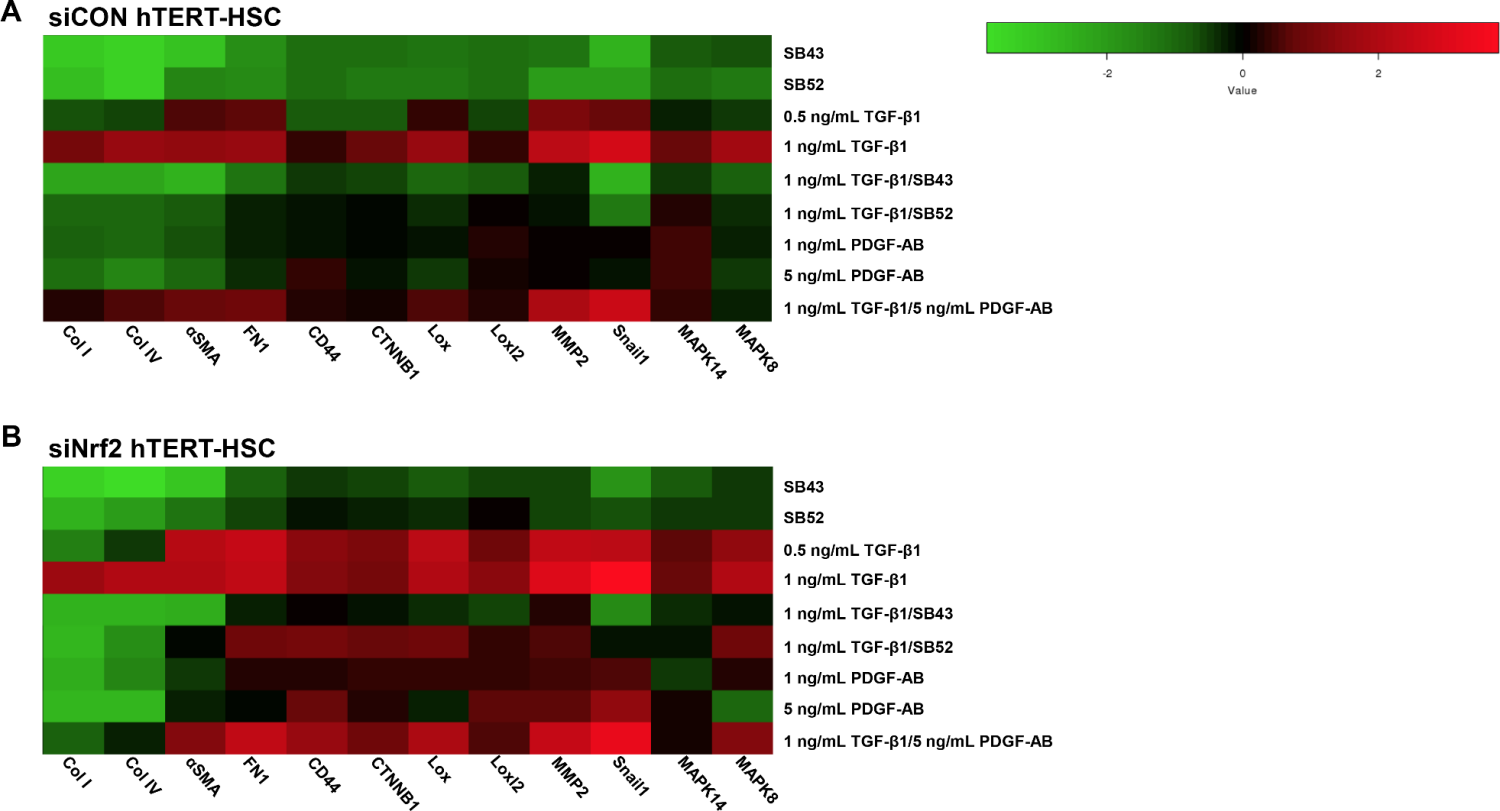
Heat map analysis of gene expression between control and Nrf2 knockdown hTERT-HSC. Heat map analysis showing differential gene expression pattern in siCON transfected hTERT-HSC (A) and siNrf2 transfected hTERT-HSC (B) Heat map was generated on -Log_2_(ΔΔCT) by using the Heatmapper online software (http://www2.heatmapper.ca/expression/). Columns represent each gene of interest, while rows indicate each cell treatment. Cells were exposure to the tested compounds for 48 hours prior mRNA extraction.

To elucidate the molecular mechanisms underneath the siNrf2-induced HSC activation, we exposed the transfected cells to TGF-β1, PDGF-AB and/or Smad inhibitors for 48 hours and assessed gene expression of activation and fibrotic markers (Fig 6). In both siCON and siNrf2 hTERT-HSC, SB43 displayed a strong inhibitory effect and decreased the TGF-β1 elicited activation of the cells. The effect of SB52 was similar but less marked. Nrf2 knockdown cells also showed higher response to TGF-β1 than siCON cells, leading to a significant upregulation of all the markers (except collagens) already at 0.5 ng/mL TGF-β1 (Fig 6B). PDGF-AB slightly induced CD44, Lox genes, CTNNB1, MMP2 and Snail1 only in siNrf2 cells. Consistently, the combination of TGF-β1 and PDGF-AB showed stronger gene expression changes in siNrf2 samples than siCON samples (Fig 6). Interestingly, SB43 successfully inhibited the TGF-β1-induced gene induction, with a weaker effect on the siNrf2 cells. SB52, showed a strong inhibitory effect on siCON HSCs, while some of the markers (FN1, CD44, CTNNB1, Lox, Loxl2, MMP2, MAPK8) were still slightly upregulated after TGF-β1/SB52 treatments. These data indicate an anti-activation and anti-fibrotic role of Nrf2 in HSCs.

## Discussion

Recently, Nrf2 has drawn attention to its role as an antifibrotic agent in the liver [17]. However, although Nrf2 has been identified as an important factor in HSC activation [3], the mechanism through which it acts in HSC remains unclear. In this study, we focused on Nrf2 pathway in both quiescent and activated HSC. Both primary and immortalised HSCs showed high level of Nrf2 and especially of Nqo1 prior treatments (Fig 1), which is consistent with previously published data on rat HSCs showing high Nrf2 content in quiescent cells and lower levels after HSCs activation [3]. These high levels of Nrf2 may contribute to maintain HSCs in their quiescent phenotype, as shown by the low level of αSMA before applying the treatments (Fig 3C and 3D). In our study, we demonstrated that TGF-β1-induced HSC activation significantly reduced the mRNA levels of both Nrf2 and Nqo1 in a concentration and time-dependent manner in human HSCs. However, TGF-β1 exposure did not significantly affect the protein levels of Nrf2 or Nqo1. A potential explanation for the changes in the mRNA level may be due to a loss of Nrf2 function rather than a decrease in protein quantity. Consistent with this hypothesis, we detected an upregulation of ATF3 after TGF-β1 exposure (S1D Fig). It has been reported that TGF-β1-induced ATF3 binds Nrf2, resulting in a strong repression of an ARE reporter, without directly affecting the Nrf2 protein level [24,25]. Nrf2 may also be anchored to the cytoskeleton by Keap1, as suggested by the increase in Keap1 protein amount after TGF-β1 exposure (Fig 1E and 1F).

Our results strongly support the direct involvement of Nrf2 in limiting HSC activation. We could show that decreased levels of Nrf2 induced a significant upregulation of genes involved either in HSC activation (αSMA, Lox, Loxl2) or ECM remodelling (Collagens I and IV) (Fig 3A and 3B). The upregulation of TGF-β1 and PDGFRB are relevant hallmarks of HSC activation, due to the involvement of these proteins in maintaining a positive loop towards activation [23,26]. Primary HSCs displayed a stronger response to siNrf2 based on the stronger upregulation of marker genes.

Decreased levels of Nrf2 in HSC also led to increased production and release of TGF-β1-levels suggesting that the HSC activation was at least partly TGF-β1/Smad-dependent. In support of this theory, the two Smad inhibitors SB43 and SB52 efficiently reduced the TGF-β1-induced increase in αSMA production and the induction of mRNA levels of most of the activation markers in siNrf2 HSCs (Fig 3C and 3D and Fig 6). The less marked activation phenotype observed with the inhibitors alone may be due to the blocking of basal activation of the Smad pathway (e.g. through endogenous TGF-ß1). On the other hand, Nrf2 accumulation in siKeap1-HSCs led to a milder activation by TGF-β1, based on induction of αSMA (Fig 3F). These results indicate that the high amount of Nrf2 in quiescent HSCs is essential to maintaining a repressed phenotype, suggesting a new role of Nrf2 as anti-fibrogenic factor in HSCs.

Migration and proliferation are important hallmarks of activated HSC as well as of cancer progression in many tissues, and they have been often related to Nrf2 pathway [1,27–30]. Here we report that Nrf2 inhibits migration and induces proliferation in quiescent HSCs. The depletion of Nrf2 significantly increased cell motility, while Keap1 knockdown significantly reduced motility in primary HSCs (Fig 5). Cell migration was not correlated to cell proliferation, as indicated by the decrease in the proliferation rate of the siNrf2 cells (Fig 4). No release of PDGF-AB was detected in either HSCs, indicating that the effects on migration and proliferation are not directly correlated to PDGF (data not shown). Similarly, the two Smad inhibitors SB43 and SB52 did not affect neither proliferation nor migration of HSCs, suggesting a Smad-independent regulatory mechanism of these two cellular processes (data not shown). Nrf2 has been shown to interact with the PI3K-AKT signalling pathway and NF-kB in regulating antioxidant- as well as proliferation- and migration-related genes in many tissues [31–33]. The antagonistic effect of Nrf2 to NF-kB may be one of the mechanisms through Nrf2 inhibits migration and invasiveness in the HSCs, as it has been shown for human embryonic kidney cells [33]. Similarly, an interaction with AKT may be acting in HSCs and regulates proliferation. AKT has been shown to play an important role in the early activation of HSC and in their protection against apoptosis [34,35]. On the other hand, the promotion of proliferation operated by Nrf2 may be correlated to its inhibitory effect on the TGF-β1 pathway, as the anti-proliferative effect of this cytokine has been widely documented on smooth muscle cells and epithelial cells [36–39]. Concordantly, our results show that TGF-β1 decreased HSC proliferation of the HSC-line and that this response was further enhanced in Nrf2-deficient cells. Depletion of Nrf2 also led to a more pronounced PDGF-AB-induced proliferation, whereas only minor effects on cell migration were observed (Fig 4C and 4D and Fig 5C).

**Fig 7:**
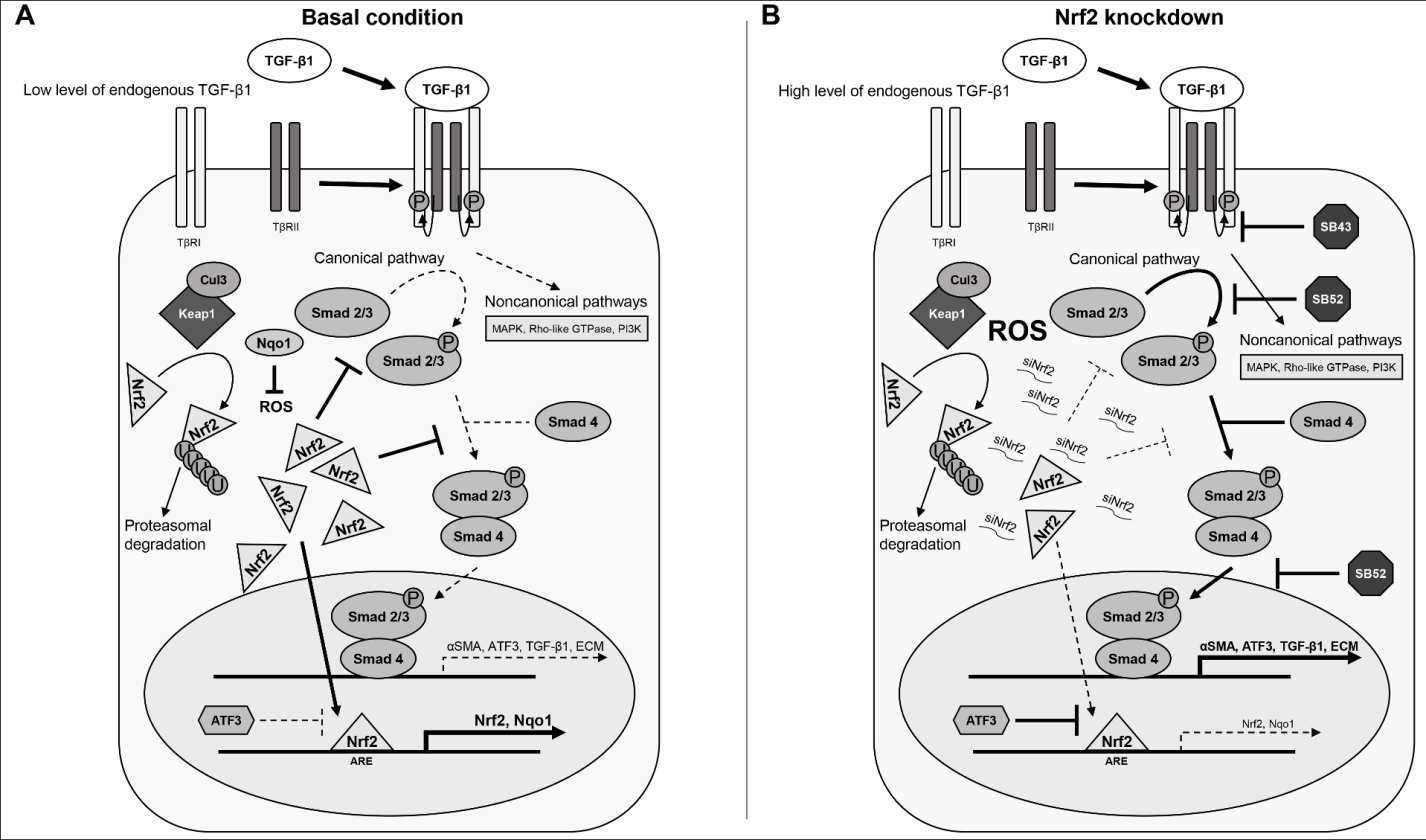
Nrf2 intervention into the TGF-β1/Smad signalling pathway. Panel A, basal condition: HSCs express high levels of both Nrf2 and its target genes (e.g. Nqo1), thereby controlling reactive oxygen species (ROS) level. Nrf2 also inhibits Smad pathway by binding directly to Smad protein or through the action of phosphatases [41]. In these conditions, the TGF-β1/Smad pathway has a low activity, resulting in low level of αSMA, collagens and TGF-β1. Thus, HSC cells exhibit a quiescent phenotype. Panel B, Nrf2 knockdown: we have found out that Nrf2 knockdown, with a consequent decrease of its target genes, induces stellate cells activation. Decrease in Nrf2 was in fact associated with an increase in the levels of Extracellular matrix (ECM) components as well as αSMA and TGF-β1. TGF-β1 further induces the expression of ATF3, which acts as functional repressor of Nrf2. We found out that this siNrf2-induced stellate cell activation may be regulated by the Smad inhibitors SB-431542 hydrate (SB43) and SB-525334 (SB52), confirming the role of Nrf2 in relation to the Smad pathway.

Our evidence provides insight into a novel role of Nrf2 in HSCs. Fig 7 illustrates the Nrf2 signalling pathway and its interplay with the TGF-β1/Smad pathway. We showed high level of both Nrf2 and Nqo1 prior TGF-β1 induction in HSCs. Nrf2, as well as Nqo1, have been identified as the most important genes involved in the oxidative response against reactive oxygen species (ROS) [40]. The high amount of Nrf2 in the HSCs may inhibit the Smad2/3 protein by inducing the gene expression of phosphatases (such as PPM1A), which may reduce the phosphorylation of Smad2/3 as previously published [41]. This would result in a low amount of active Smad2/3, favouring a proliferative status rather than the induction of the genes involved in HSC activation and motility. This is concordant with the phenotype of control HSCs observed in our experiments, characterized by low levels of activation markers (αSMA and TGF-β1). Nrf2 has also been shown to bind Smad proteins in cancer cell lines, acting as a transcriptional repressor by competing with Smad complex for the co-transcriptional activator p300/CBP [27]. These observations point out Nrf2 as a key factor in maintaining a repressed phenotype in HSC, which is in agreement with the activation elicited in the Nrf2 knockdown experiments.

Following Nrf2 knockdown (Fig 7B), Nqo1 levels in the cytoplasm decrease quickly, which may result in an increase of ROS and its inhibitory effect of PPM1A [41]. Thus, Smad2/3 could bind Smad4 and translocate to the nucleus, resulting in a strong induction of HSC activation markers, induction of migration and inhibition of proliferation. In line with this hypothesis, Smad inhibitors could modulate siNrf2-induced HSC activation, confirming the involvement of the TGF-β1/Smad pathway. Interestingly, SB43 showed a stronger inhibition than SB52, both alone and in combination with TGF-β1. This may be related to their different inhibition mechanism. SB43 has been shown to inhibit the TGF-β type I receptor (TβRI), acting more upstream on the pathway compared to SB52, which inhibits Smad3 phosphorylation and nuclear translocation [42–44]. Nrf2 knockdown (and TGF-β1 release), as well as the addition of exogenous TGF-β1, lead to high amount of phosphorylated Smad2/3 which implies SB52 to be ineffective at fully repressing TGF-β1-induced gene expression. New synthesized ATF3 could further contribute to the Nrf2 inhibition, exacerbating more the TGF-β1-induced HSC activation.

In conclusion, our data provide clear proof of the direct involvement of Nrf2 in HSC activation. This underlines the importance of Nrf2 in non-parenchymal liver cells in addition to its already known cytoprotective role in hepatocytes. Our data also provide insights into the mechanisms by which Nrf2 decreases HSC activation, highlighting a new role of Nrf2 as anti-fibrotic molecule in HSCs. Indeed, the depletion of Nrf2 may be a contributing factor to HSC activation *in vitro* as well as *in vivo*. Intervention on Nrf2 pathway in HSC may be a new perspective therapy for liver fibrosis.

## Supporting information

S1 Fig TGF-β1 effects on Nrf2 pathway’s component. (A-C) hTERT-HSCs were exposed to 1–5 ng/mL TGF-β1 for 24, 48 and 72 hours. mRNA was extracted using TRIzol conventional procedure and fold changes were calculated as 2^(-ΔΔCT) for each sample and control and expressed as mean fold change ± SD (N = 3). Beta-2-microglobulin (B2M) was used as reference gene for each sample. The results show a significant downregulation of Nrf2, Keap1 and Nqo1 after exposure to TGF-β1 in a time- and concentration-dependent manner. (A) Nrf2 mRNA levels; (B) Keap1 mRNA levels; (C) Nqo1 mRNA levels. (D) mRNA levels of ATF3 were analysed in both hTERT-HSC and primary HSC after exposure to 1 ng/mL TGF-β1 for 48 hours. Fold induction were calculated as 2^(-ΔΔCT) for each sample and control and expressed as mean fold induction ± SD (N = 3 for hTERT-HSC and N = 5 different batches for primary HSC). *, P ≤ 0.05; **, P ≤ 0.01; ***, P ≤ 0.001 vs Control.
(TIF)

## Abbreviations

hTERT-HSC: Immortalised hepatic stellate cells
SB43: SB-431542 hydrate
SB52: SB-525334
siCON: scrambled siRNA
siNrf2: siRNA targeting Nrf2
siKeap1: siRNA targeting Keap1
