# Supplementary figures and images for: Nrf2 protects stellate cells from Smad-dependent cell activation
Source: PLoS One. 2018 Jul 20;13(7):e0201044. doi: 10.1371/journal.pone.0201044 (PMC6054401; doi:10.1371/journal.pone.0201044)

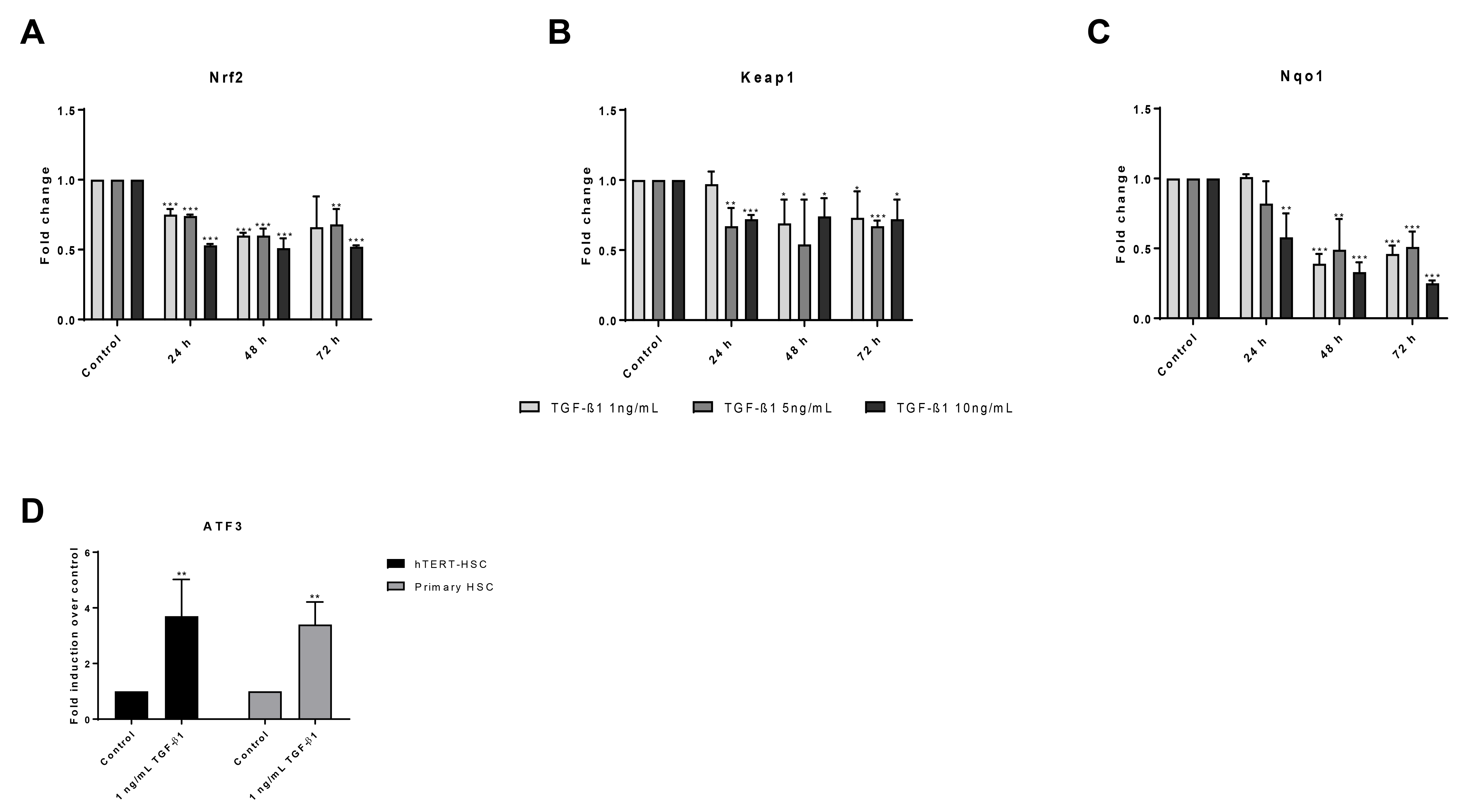

Supplement: S1 Fig — (A-C) hTERT-HSCs were exposed to 1–5 ng/mL TGF-β1 for 24, 48 and 72 hours. mRNA was extracted using TRIzol conventional procedure and fold changes were calculated as 2^(-ΔΔCT) for each sample and control and expressed as mean fold change ± SD (N = 3). Beta-2-microglobulin (B2M) was used as reference gene for each sample. The results show a significant downregulation of Nrf2, Keap1 and Nqo1 after exposure to TGF-β1 in a time- and concentration-dependent manner. (A) Nrf2 mRNA levels; (B) Keap1 mRNA levels; (C) Nqo1 mRNA levels. (D) mRNA levels of ATF3 were analysed in both hTERT-HSC and primary HSC after exposure to 1 ng/mL TGF-β1 for 48 hours. Fold induction were calculated as 2^(-ΔΔCT) for each sample and control and expressed as mean fold induction ± SD (N = 3 for hTERT-HSC and N = 5 different batches for primary HSC). *, P ≤ 0.05; **, P ≤ 0.01; ***, P ≤ 0.001 vs Control. (TIF) [file pone.0201044.s001.tif]
